# Hsa_circ_0028502 and hsa_circ_0076251 are potential novel biomarkers for hepatocellular carcinoma

**DOI:** 10.1002/cam4.2584

**Published:** 2019-10-08

**Authors:** Zhenluo Jiang, Lili Shen, Shuwei Wang, Shengdong Wu, Yaoren Hu, Junming Guo, Liyun Fu

**Affiliations:** ^1^ Department of Hepatology HwaMei Hospital University Of Chinese Academy Of Sciences Ningbo China; ^2^ Department of Biochemistry and Molecular Biology Zhejiang Key Laboratory of Pathophysiology Medical School of Ningbo University Ningbo China; ^3^ Department of General Surgery Li Hui‐Li Hospital and the Affiliated Hospital Medical School of Ningbo University Ningbo China

**Keywords:** biomarkers, has_circ_0028502, has_circ_0076251, hepatocellular carcinoma

## Abstract

Circular RNAs (circRNAs) have been increasingly revealed to be desirable biomarkers for some tumors, including hepatocellular carcinoma (HCC). Combined with our previous microarray screening results, we aimed to determine the hsa_circ_0028502 and hsa_circ_0076251 expression features in HCC, analyze the relationship between their expression level and clinical and pathological characteristics, and investigate their diagnostic and prognostic values. Our data demonstrated that the hsa_circ_0028502 and hsa_circ_0076251 levels were considerably lower in HCC tissues than in adjacent paracancerous tissues (*P* < .001). Further study revealed that hsa_circ_0028502 expression levels were related to tumor node metastasis (TNM) stage (*P* = .015) and that hsa_circ_0076251 expression levels were related to Barcelona Clinic Liver Cancer (BCLC) stage (*P* = .038), comorbidity with type 2 diabetes mellitus (*P* = .023) and the presence of serum HbsAg (*P* = .044). Furthermore, the degree of expression of both hsa_circ_0028502 and hsa_circ_0076251 increased from HCC to liver cirrhosis (LC) to chronic hepatitis (CH). The receiver operating characteristic (ROC) curve demonstrated that hsa_circ_0028502 and hsa_circ_0076251 could serve as fairly accurate markers to distinguish HCC tissues from CH tissues and LC tissues, as well as distinguishing LC tissues from CH tissues. Cox regression analysis showed that low expression of has_circ_0076251 was associated with unfavorable survival rates in HCC (HR = 0.46; 95% CI = 0.22‐0.98; *P* < .05). These findings implied that hsa_circ_0028502 and hsa_circ_0076251 were potentially valuable biomarkers for HCC diagnosis, whereas hsa_circ_0076251 could be used as a prognostic indicator for HCC.

## Graphical abstract

hsa_circ_0028502 and hsa_circ_0076251 are potentially valuable biomarkers for HCC diagnosis, and meanwhile, hsa_circ_0076251 could serve as a prognostic indicator for HCC.

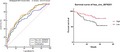

## 1 INTRODUCTION

Hepatocellular carcinoma (HCC) is fourth leading cause of death from cancer worldwide and was the second leading cause of men's deaths in 2018.^1^, ^2^ Patients who are identified at early stages can benefit from resection, locoregional therapy, or liver transplantation.^3^ However, more than 70% of patients with HCC who were screened by current diagnostic methods had advanced‐stage disease and were not amenable to effective treatment.^4^, ^5^ There is an urgent need for new biomarkers for the accurate and early diagnosis of HCC.

Unlike linear RNAs, circular RNAs (circRNAs), the 5ʹ and 3ʹ ends of which are covalently jointed in an annular structure, have been a recent focus because they have been found to play increasingly more important roles in the regulation of gene expression, such as acting as a scaffold for RNA or proteins, microRNA (miRNA) sponge, splicing regulator, etc.^6^, ^7^, ^8^, ^9^ In the last 10 years, emerging studies have demonstrated that circRNAs play a role in carcinogenesis and metastasis in many cancers; however, the roles of circRNAs in the detection and pathological process of HCC are still largely unknown.^10^, ^11^ Because of their special properties, such as conservation, stability, tissue specificity, and stage‐related expression, circRNAs can serve as promising novel targets for cancer screening and therapy evaluation.^12^, ^13^

Deriving data from our microarray screening of five paired HCC and adjacent nontumor tissues (GEO No. 94508: https://www.ncbi.nlm.nih.gov/geo/query/acc. cgi?acc=http://www.ncbi.nlm.nih.gov/geo/query/acc.cgi?acc=GSE94508), we focused on two circRNAs in this study, hsa_circ_0028502 and hsa_circ_0076251, which were found to be most downregulated in HCC tissues.^14^ Hsa_circ_0028502 is transcribed from SLC24A6 (Solute Carrier Family 8 member B1) on chromosome 12, and hsa_circ_0076251 is transcribed from ZFAND3 (Zinc Finger AN1‐type containing 3) on chromosome 6. Our results indicate that hsa_circ_0028502 and hsa_circ_0076251 may serve as novel promising biomarkers for HCC.

## 2 MATERIAL AND METHODS

### 2.1 Sample collection and ethics statement

The 100 paired samples of HCC and adjacent liver tissue were obtained from surgical patients in three hospitals—Ningbo Li Hui‐Li Hospital, HwaMei Hospital, University Of Chinese Academy Of Sciences and Ningbo Yinzhou Peoples’ Hospital—from March 2013 to January 2017. Patients with HCC who received prior therapy or had other solid tumors were not included in this study. Paired adjacent nontumorous tissue located at least 1 cm away from the margin of the HCC tissue was excised by surgeons and then confirmed by two trained pathologists to have no obvious tumor cells. We further collected 37 tissue samples from patients with chronic liver diseases from HwaMei Hospital, University Of Chinese Academy Of Sciences, through ultrasound‐guided liver biopsy from September 2013 to January 2017. All specimens were promptly preserved in RNAfixer reagent (Bioteke, Beijing, China) after removal from patients and were immediately stored at −80°C until analysis. All details of this study were approved by the Human Research Ethics Committee of Ningbo University (IRB No. 20100303). Clinical information was obtained with written consent from each participant.

HCC patients were evaluated according to the Barcelona Clinic Liver Cancer staging system (BCLC) 15 and the tumor node metastasis (TNM) staging system.^16^ The stage of liver tissue fibrosis and histological inflammatory activity was assessed by the METAVIR scoring system (the stage of fibrosis was scored according to a five‐point scale: cirrhosis = F4, numerous septa without cirrhosis = F3, portal fibrosis with rare septa = F2, portal fibrosis without septa = F1, no fibrosis = F0; the stage of histological inflammatory activity was scored according to a four‐point scale: severe inflammation = A3, moderate inflammation = A2, mild inflammation = A1, no inflammation = A0).^17^

### 2.2 Total RNA extraction

Total RNA was extracted from each sample using TRIzol Reagent (Invitrogen, Karlsruhe, Germany) per the manufacturer's detailed instructions. RNA was deemed fit for subsequent experiments if the ratio of absorbance at 260 and 280 nm was between 1.8 and 2.0.

### 2.3 Reverse transcription

cDNA was produced utilizing the GoScript Reverse Transcription (RT) System (Promega, Madison, WI, USA) based on manufacturer‐provided instructions.

### 2.4 Quantitative real‐time PCR

**Figure 1:**
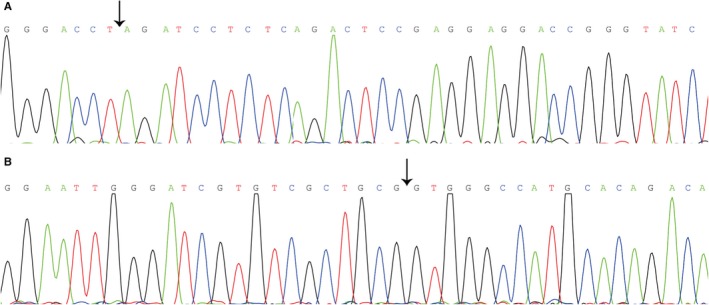
Sequence of qRT‐PCR products. A, Sequence of qRT‐PCR products of hsa_circ_0028502. B, Sequence of qRT‐PCR products of hsa_circ_0076251. Black arrow indicate the splicing junction sites

Real‐time quantitative reverse transcription‐polymerase chain reaction (qRT‐PCR) was performed with the GoTaq qPCR Master Mix (Promega) in an Mx3005P real‐time PCR system (Stratagene, La Jolla, CA, USA) according to the manufacturer's detailed protocols. Glyceraldehyde 3‐phosphate dehydrogenase (GAPDH), a housekeeping gene, was used as a control. Outward facing primers were first devised with Primer‐BLAST (https://www.ncbi.nlm.nih.gov/tools/primer-blast/) and later by Sangon Biotech (Shanghai, China), with the following sequences: 5ʹ ‐GGGACCTAGATCCTCTCAGACT‐3ʹ (sense) and 5ʹ ‐GCCGGTACTCATCACCGTAG‐3ʹ (anti‐sense) for hsa_circ_0028502; 5ʹ ‐CGGCCACGACTACTTGAGAA‐3ʹ (sense) and 5ʹ ‐ACTGTGAATCTGTACCACAGGA‐3ʹ (anti‐sense) for hsa_circ_0076251; 5ʹ ‐TCGACAGTCAGCCGCATCTTCTTT‐3ʹ (sense) and 5ʹ ‐ACCAAATCCGTTGACTCCGACCTT‐3ʹ (anti‐sense) for GAPDH. The single peak in the melting curve appeared suggested the specificity of the PCR products. The head‐to‐tail splicing sites of hsa_circ_0028502 and hsa_circ_0076251 are verified through cloning and sequencing of qRT‐PCR products (Figure 1). The expression data were analyzed using the △C*t* method, and lower values denote high expression levels. The results are expressed as the mean ± standard deviation (SD) based on three repeated independent experiments. All assays were performed in a blinded manner.

### 2.5 Sequencing of qRT‐PCR products

Following the manufacturer's instructions, the qRT‐PCR products of hsa_circ_0028502 and hsa_circ_0076251 were purified using a UNIQ‐10 PCR Product Purification Kit before being cloned into the pUCm‐T vector (Sangon Biotech). Thereafter, RNA sequencing was conducted by Sangon Biotech Co., Ltd.

### 2.6 Statistical analysis

Statistics were analyzed using GraphPad Prism 7.0 (GraphPad Software, La Jolla, CA, USA), the Statistical Product and Service Solutions (SPSS) 18.0 software package (IBM, Chicago, IL, USA), and MedCalc software (MedCalc software bvba, Ostend, Flanders, Belgium). In this study, paired *t* test, independent *t* test, and one‐way analysis of variance (ANOVA) were used. The diagnostic power was studied by receiver operating characteristic (ROC) curve. Through the use of log‐rank and Cox regression analyses, differences in patient overall survival were evaluated. *P* values of .05 or less were considered statistically significant.

## 3 RESULTS

### 3.1 Downregulation of hsa_circ_0028502 and hsa_circ_0076251 expression in HCC tissues

To screen for differentially regulated circRNAs profiles, human circular RNA microarray analysis was used to study five pairs of HCC tissue and adjacent nontumorous tissue.^14^ Hundreds of differentially expressed circRNAs were discovered. In combination with circBase, we chose two significantly downregulated and middle‐ranking circRNAs, hsa_circ_0028502 (36.13‐fold difference) and hsa_circ_0076251 (33.48‐fold difference), for our further study.

**Figure 2:**
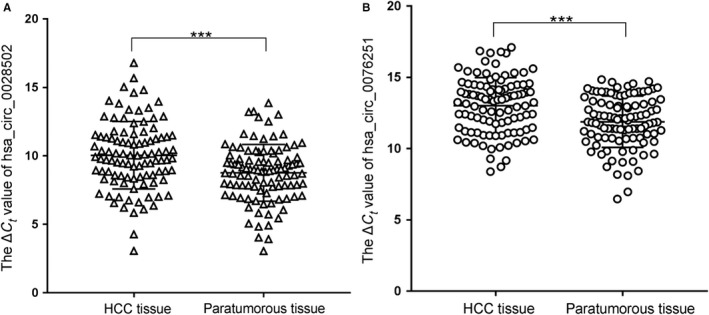
Hsa_circ_0028502 and hsa_circ_0076251 expression features in HCC tissues. A, Hsa_circ_0028502 levels in 100 HCC tissues and their matched adjacent nontumorous tissues were detected using qRT‐PCR method. B, Hsa_circ_0076251 levels in 100 HCC tissues and their matched adjacent nontumorous tissues were detected using qRT‐PCR method. Larger ΔCt value indicates lower expression. Data are means ± SD. ***P<.001

To verify the preliminary results of our microarray, we expanded the sample size to 100 paired samples from surgically resected HCC and adjacent nontumorous tissues. The relative expression levels of hsa_circ_0028502 and hsa_circ_0076251 were subjected to qRT‐PCR analysis, and the results show that hsa_circ_0028502 and hsa_circ_0076251 were both downregulated in HCC tissues relative to adjacent nontumorous tissues (Figure 2). Our results showed that the expression properties of hsa_circ_0028502 and hsa_circ_0076251 in a large sample size were consistent with the results of our microarray.

### 3.2 Relationships between circRNA (has_circ_0028502 and has_circ_0076251) expression and clinical pathological factors in HCC

As above, we have found that has_circ_0028502 and has_circ_0076251 expression levels were downregulated in HCC tissue. Then, we examined our data for potential correlations between has_circ_0028502 and has_circ_0076251 expression levels and clinical pathological parameters. As shown in Table 1, we found that TNM stage (*P* = .015) was related to the level of hsa_circ_0028502 expression. In addition, the hsa_circ_0076251 level was related to comorbidity with type 2 diabetes mellitus (DM) (*P* = .023), BCLC stage (*P* = .038) and the presence of hepatitis B surface antigen (HbsAg) (*P* = .044).

**Table 1:** Correlations between circRNA (has_circ_0028502, has_circ_0076251) expression levels and clinical parameters in HCC

Characteristics	Patient number	has_circ_0028502		has_circ_0076251	
	(n)	Mean ± SD	*P* value	Mean ± SD	*P* value
Age (years)					
≥50	76	10.11 ± 2.41	.65	13.01 ± 1.97	.911
<50	24	9.85 ± 2.66		13.07 ± 1.97	
Gender					
Male	86	9.91 ± 2.48	.157	12.93 ± 1.91	.216
Female	14	10.92 ± 2.26		13.63 ± 2.22	
Family history					
Positive	59	9.83 ± 2.56	.283	12.81 ± 2.07	.188
Negative	41	10.37 ± 2.31		13.34 ± 1.76	
T2 DM					
Yes	14	9.78 ± 2.93	.647	11.93 ± 2.33	.023
No	86	10.10 ± 2.39		13.21 ± 1.85	
Encapsulation					
Yes	66	10.08 ± 2.27	.868	13.03 ± 2.01	.953
No	33	9.99 ± 2.88		13.00 ± 1.91	
Tumor number					
Single	67	10.33 ± 2.43	.107	12.95 ± 1.82	.64
Multiple	32	9.47 ± 2.50		13.15 ± 2.27	
Diameter(cm)					
≥5	44	10.12 ± 2.43	.825	13.02 ± 1.80	.992
<5	55	10.00 ± 2.53		13.02 ± 2.11	
Differentiation					
Poor	14	10.00 ± 2.69	.987	12.60 ± 1.85	.39
Moderate and well	83	10.01 ± 2.43		13.08 ± 1.93	
Microvascular invasion					
Positive	39	9.94 ± 2.63	.778	12.55 ± 1.74	.061
Negative	56	10.09 ± 2.39		13.30 ± 1.99	
BCLC stage					
A + B	82	10.09 ± 2.40	.772	12.83 ± 1.91	.038
C + D	17	9.89 ± 2.89		13.92 ± 2.05	
TNM stage					
Ⅰ+Ⅱ	68	10.27 ± 2.79	.015	12.83 ± 2.03	.157
Ⅲ+Ⅳ	31	11.72 ± 2.67		13.43 ± 1.79	
HbsAg					
Positive	79	10.04 ± 2.41	.34	13.23 ± 1.91	.044
Negative	19	10.17 ± 2.83		12.21 ± 2.08	
Serum AFP					
>20	54	10.23 ± 2.31	.443	12.96 ± 2.03	.741
≤20	43	9.84 ± 2.13		13.09 ± 1.94	
Serum AKP					
>95	55	9.92 ± 2.60	.693	13.01 ± 1.99	.997
≤95	44	10.12 ± 2.26		13.01 ± 1.95	
Serum GGT					
>50	55	9.77 ± 2.54	.269	12.74 ± 2.05	.131
≤50	44	10.31 ± 2.31		13.34 ± 1.88	

Abbreviation: AFP, α‐fetoprotein; AKP, alkaline phosphatase; BCLC, barcelona clinic liver cancer staging system; GGT, gamma glutamyl transferase; HbsAg, hepatitis B antigen; T2 DM, type 2 diabetes mellitus; TNM, tumor node metastasis.

### 3.3 Differential expression levels of hsa_circ_0028502 and hsa_circ_0076251 in CH, LC, and HCC tissues

**Figure 3:**
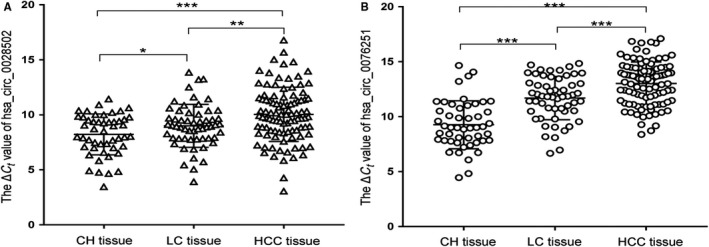
Hsa_circ_0028502 and hsa_circ_0076251 expression features in different liver tissues. A, Decreased expression levels of hsa_circ_0028502 from chronic hepatitis (CH) to liver cirrhosis (LC) to hepatocellular carcinoma (HCC). B, Decreased expression levels of hsa_circ_0076251 from CH to LC to HCC. Data are mean ± SD. *P<.05, **P<.01, ***P<.001

The 70 cases of adjacent nontumorous liver tissues were pathologically diagnosed according to the METAVIR scoring system; among them, 56 cases of liver cirrhosis (LC) were diagnosed (F = 4), along with 14 cases of chronic hepatitis (CH) (F = 0‐3). Moreover, we collected 36 samples of liver tissue from hepatitis patients by ultrasound guided biopsy. Among them, one sample showed LC (F = 4), and 35 samples were diagnosed with CH (F = 0‐3). Consequently, the LC group is composed of 56 paratumorous liver tissue samples and 1 hepatitis liver tissue sample that were found to be METAVIR stage F4 regardless of the stage of histological inflammation activity. The CH group is composed of 14 paratumorous liver tissues samples and 35 hepatitis liver tissue samples that were found to be METAVIR stage F0‐3 regardless of the stage of histological inflammation activity. The relative expression levels of hsa_circ_0028502 in LC tissue were lower than those in CH tissue (*P* < .05), and the relative expression levels of hsa_circ_0028502 in HCC tissues were lower than those in LC tissues (*P* < .01), as shown in Figure 3A. The relative expression levels of hsa_circ_0076251 in LC tissues were also lower than those in CH tissue (*P* < .001), and the relative expression levels of hsa_circ_0076251 in HCC tissue were found to be lower than those in LC tissues (*P* < .001), as shown in Figure 3B.

### 3.4 Potential use of hsa_circ_0028502 and has_circ_0076251 expression levels as biomarkers for diagnosis

**Figure 4:**
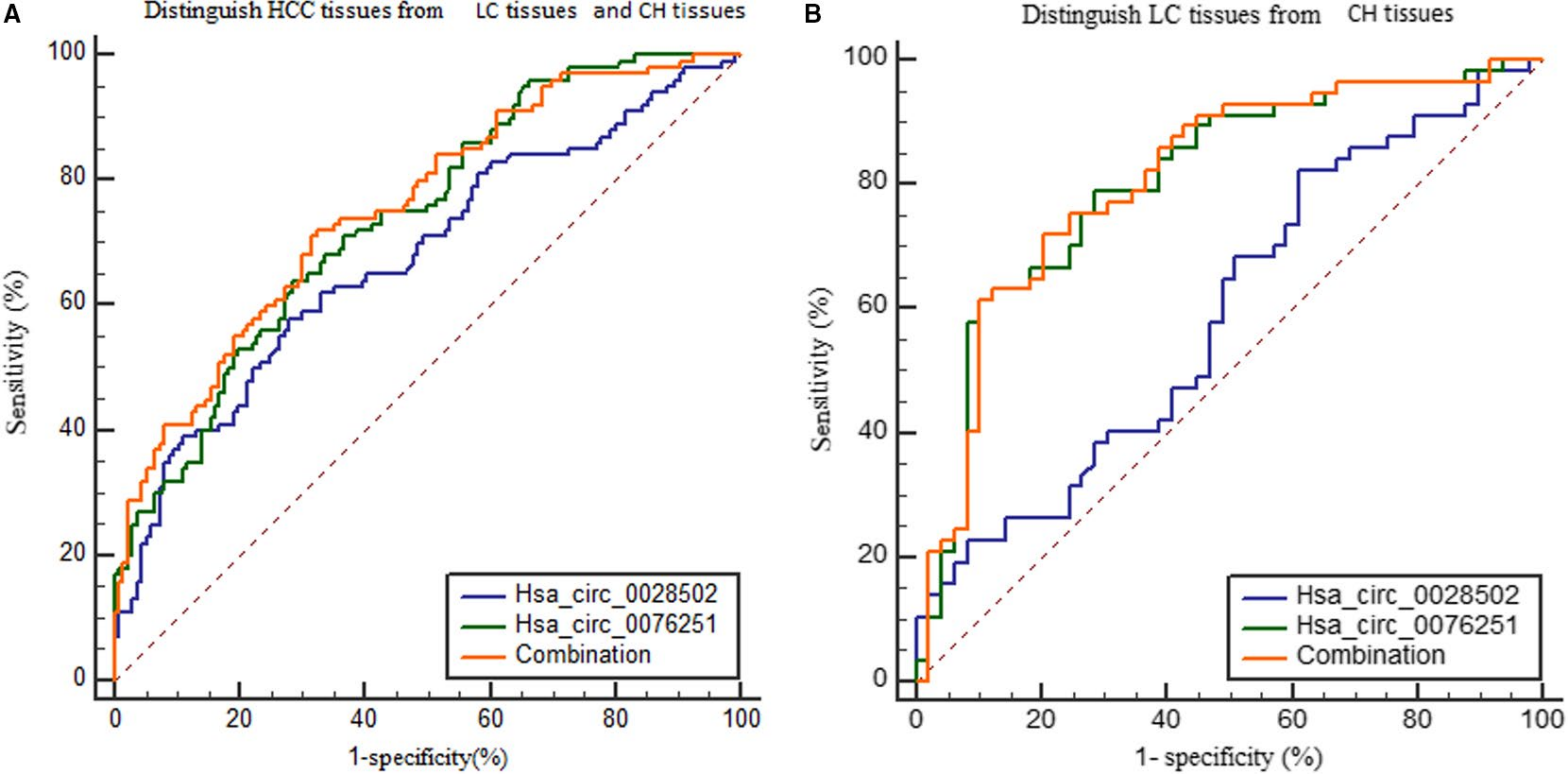
Diagnostic value of hsa_circ_0028502 or has_circ_0076251 alone, and the combination of these two markers for the detection of HCC. A, Distinguish HCC tissues from LC tissues and CH tissues. B, Distinguish LC tissues from CH tissues

ROC curves were plotted to assess the diagnostic values of hsa_circ_0028502 and has_circ_0076251 expression levels. Hsa_circ_0028502 was used to distinguish HCC tissues from LC tissues and CH tissues (AUC = 0.675, specificity = 0.721, sensitivity = 0.580, and cut‐off value = 9.595); hsa_circ_0076251 was used to distinguish HCC tissues from LC tissues and CH tissues as well, but with different values (AUC = 0.738, specificity = 0.713, sensitivity = 0.640, and cut‐off value = 12.340). Furthermore, these two indicators could distinguish LC tissue from CH tissue (AUC = 0.592, specificity = 0.388 sensitivity = 0.825, and cut‐off value = 7.720 for hsa_circ_0028502; AUC = 0.803, specificity = 0.898, sensitivity = 0.614, and cut‐off value = 11.410 for hsa_circ_0076251). We then analyzed the combined diagnostic value of these two indicators by multiple linear regression analysis. The efficiency of the combined diagnosis in distinguishing HCC cases from LC cases and CH cases (AUC = 0.754, specificity = 0.676, and sensitivity = 0.720) and LC cases from CH cases (AUC = 0.807, specificity = 0.796, and sensitivity = 0.719) was higher than the efficiency of using hsa_circ_0028502 or hsa_circ_0076251 expression levels alone (Figure 4).

### 3.5 Correlations between hsa_circ_0028502 and has_circ_0076251 with survival in HCC

**Figure 5:**
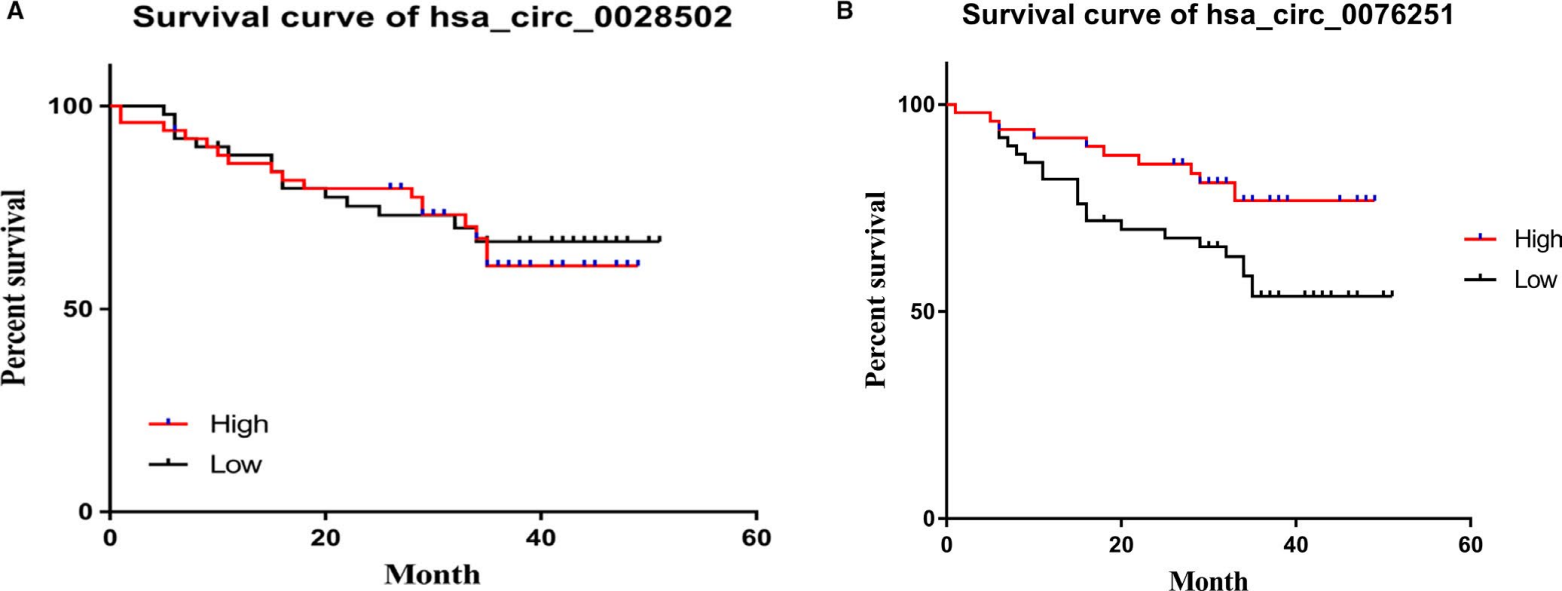
Survival curves of has_circ_0028502 and has_circ_0076251. A, Survival curves of has_circ_0028502. B, Survival curves of has_circ_0076251

To explore the potential correlations between has_circ_0028502 and has_circ_0076251 expression levels and the outcome of HCC patients, we categorized enrolled patients into high and low expression groups of has_circ_0028502 (or has_circ_0076251) according to the median value. Based on follow‐up data, the survival curves of the two groups of has_circ_0076251 expression were analyzed by the log‐rank test using univariate analysis (*P* < .05) (Figure 5). We then used multivariate Cox regression analysis to assess the prognostic values of some clinical pathological factors, including age, gender, TNM stage, BCLC stage, and has_circ_0076251 expression. The results showed that low expression of has_circ_0076251 is connected to unfavorable survival in HCC patients (HR = 0.46; 95% CI = 0.22‐0.98; *P* < .05). This result suggested that has_circ_0076251 could act as an independent prognostic marker for HCC.

## 4 DISCUSSION

CircRNAs were first observed to naturally exist in plant viroids.^18^, ^19^ They were considered to have no biological function until the past few years.^20^ Due to advances in genomics and high‐throughput sequencing technologies, an increasing number of circRNAs have been screened recently.^21^, ^22^ CircRNAs are produced mainly through a type of alternative RNA splicing called “back‐splicing,” in which a downstream splice donor is connected to an upstream splice acceptor via splice skipping or direct splice.^23^ Currently, there is increasing evidence demonstrating that circRNAs are endogenous, stable, abundant, and conserved in mammalian cells.^12^ A large number of studies in recent years at home and abroad have found that circRNAs could act as novel biomarkers. For example, Qin et al^24^ reported that hsa_circ_0001649 is a novel biomarker for HCC with high degrees of sensitivity and specificity. Shang et al^25^ showed that hsa_circ_0005075 could be used to diagnose HCC. Huang et al^26^ discovered that circRNA‐100338 could serve as a prognostic indicator for HCC. KOU et al^27^ demonstrated that hsa_circ_0078602 expression level was linked to prognosis among patients with HCC.

In our preliminary study, we used circRNA microarray analysis to detect the circRNAs expression profiles in five pairs of HCC tissue and the corresponding adjacent tissue, and we found hundreds of differentially expressed circRNAs.^14^ From them, we selected two significantly differentially expressed circRNAs, hsa_circ_0028502 and hsa_circ_0076251, for our study. We then found that hsa_circ_0028502 and hsa_circ_0076251 were both markedly downregulated in HCC tissues compared to adjacent nontumorous liver tissues (Figure 2). Furthermore, we emphasize that the expression levels of hsa_circ_0028502 are connected to TNM stage and that hsa_circ_0076251 was associated with the comorbidities of diabetes mellitus (DM), BCLC stage and presence of HbsAg (Table 1).

HCC mainly develops from cirrhosis, which mostly results from chronic hepatitis B virus (HBV) infection in Asia.^28^, ^29^, ^30^ High HBV infection prevalence may result in a high incidence of hepatocarcinogenesis compared with a population without infection.^31^ As is shown in Table 1, the infection rate of hepatitis B virus was as high as 80.61% (79/98) in our study, further illustrating that hepatitis B virus infection is known to be a major risk factor for HCC. It is well‐known that LC is significantly associated with HCC.^32^ Seventy cases of adjacent nontumorous liver tissues were enrolled in this study, among which 56 cases were diagnosed with LC, or 80%. This result indicates that LC occurs prior toHCC, consistent with theories of the tumor microenvironment. The expression levels of both hsa_circ_0028502 and hsa_circ_0076251 decreased successively from CH to LC to HCC. This result indicated that hsa_circ_0028502 and hsa_circ_0076251 may have different expression characteristics in different stages of liver disease and are contributors to HCC development.

DM is characterized by abnormal glucose metabolism caused by the relative or absolute deficiency of insulin.^33^ DM is mainly divided into type 1 (T1DM), type 2 (T2DM) and gestational diabetes (GDM). Epidemiological evidence suggests that DM is one of the risk factors for carcinogenesis and poor prognosis in multiple cancers, including HCC.^34^, ^35^ The mechanisms of DM and carcinogenesis include several aspects, such as hyperinsulinemia, hyperglycemia, and chronic inflammation.^36^ We preliminarily revealed that the expression level of hsa_circ_0076251 is associated with whether the HCC patient is suffering from DM or not. The expression level of hsa_circ_0076251 was high in the HCC group with T2DM but low in the non‐T2DM group. Singh et al^37^ reported that anti‐diabetic medications (ADMs) could influence the risk of HCC in patients with DM. Therefore, DM treatments may affect the expression level of hsa_circ_0076251, which needs further experiments to be validated.

For decades, the combination of serum alpha‐fetoprotein (AFP) and ultrasound surveillance was the most commonly used means for screening for HCC in high‐risk populations.^38^ Nevertheless, ultrasound surveillance often leads to missed diagnosis of small tumors.^39^ Meanwhile, because of inadequate sensitivity and the positive predicative value of AFP, AFP determination is also far from being satisfactory.^39^, ^40^ In our recruited HCC group, the AFP‐positive rates were approximately 55.7% (54/97) using a threshold of 20 ng/mL and were close to the previous reported level.^39^ Thus, there is an urgent need for novel biomarkers for HCC. ROC analysis indicated that hsa_circ_0076251 and hsa_circ_0028502 had better value for distinguishing not only HCC from LC and CH but also LC from CH. Surprisingly, by combining hsa_circ_0076251 and hsa_circ_0028502, we found that the sensitivity of diagnosis was higher (0.720) than that of hsa_circ_0028502 (0.580) or hsa_circ_0076251 (0.640) alone (Figure 4).

Because of the high frequency of recurrence and metastasis, HCC patients’ 5‐year survival rate remains dismal.^41^, ^42^ For this reason, there is an urgent need for potential biomarkers for prognosis predication. Our research showed that high expression of has_circ_0076251 was related to favorable survival rates in HCC (Figure 5). Accordingly, different therapeutic regimens might be tailored for HCC patients with different expression levels of hsa_circ_0076251 after determining the patient's prognosis.

In conclusion, we discovered two new circRNA‐based biomarkers for the diagnosis of HCC with a high degree of accuracy, specificity, and sensitivity. The results showed that hsa_circ_0028502 and hsa_circ_0076251 were both downregulated in HCC. In addition, they both have stage‐specific expression features in different liver diseases. Further research demonstrated that has_circ_0076251 might be a prognostic marker. However, there are also some weaknesses in this article. First, a study with a greater number of samples should be conducted to verify our findings in the future so that the results can be applied to clinical practice for the diagnosis and prognosis of patients with HCC. Second, we should try to use serum circRNAs as biomarkers, making this method more feasible and reasonable.

## CONFLICT OF INTEREST

The authors made no disclosures.

## ETHICAL APPROVAL

This study was approved by the Human Research Ethics Committee of Ningbo University School of Medicine (IRB No.20100303).
